# Perceptions of college students in consuming whole grain foods made with Brewers’ Spent Grain

**DOI:** 10.1002/fsn3.872

**Published:** 2018-11-29

**Authors:** Shannon Combest, Cynthia Warren

**Affiliations:** ^1^ Department of Nutrition and Food Sciences Texas Woman's University Denton Texas

**Keywords:** Brewers’ Spent Grain, focus groups, perceptions

## Abstract

One‐third of all food produced for human consumption is wasted producing landfill accumulation and greenhouse gas emissions. Brewers’ Spent Grains (BSGs) are the leftover grains from beer production, and each year approximately 30 million tons of BSG is generated globally by the brewing industry. Reclaiming BSG as a potential human food source is an opportunity for reducing food waste in the food supply chain. Six focus groups were conducted using 37 college students to determine their consumption of whole grains, perceptions of whole grains versus refined grains, and interest in or barriers related to consuming and purchasing foods made with BSG. Focus groups were transcribed verbatim and analyzed using constant comparative analysis to identify themes and discover relationships among the study aims. Thirteen themes emerged from focus group discussions with Concept of Health, Sensory, and Experience with BSG representing the top three discussed. Participants believed whole grains are healthier and contain more nutrients than refined grains. Most participants enjoyed the BSG foods provided; however, some noted a darker appearance and lingering fiber particles or aftertaste. Findings indicate participants who are hereditary whole grain consumers are acculturated to whole grain sensory attributes and nutritional benefits and would be more receptive to consuming BSG foods in future studies. We concluded most focus group participants were open to tasting BSG foods, but hereditary whole grain consumers should be the target consumer audience, and educating consumers on sensory attributes, potential health benefits, and environmental benefits is necessary to overcome the barriers associated with BSG.

## 1 INTRODUCTION

Barley (*Hordeum vulgare*) is a whole grain used in Western countries predominantly for animal feed and malting. However, there is growing interest in barley for human diets due to its associated health benefits, including improvement of risk factors associated with cardiovascular disease and type 2 diabetes through cholesterol lowering (AbuMweis, Jew, & Ames, 2010; Talati, Baker, Pabilonia, White, & Coleman, 2009; Tiwari & Cummins, 2011) and reducing the glycemic response (Poppitt, van Drunen, McGill, Mulvey, & Leahy, 2007; Yean Soong, Yu Chin Quek, & Henry, 2015). Beta‐glucan (β‐glucan) is a soluble fiber and long‐chain polysaccharide with D‐glucose monomers connected through (1,3) and (1,4) β‐glycosidic bonds found mainly within the endosperm of the kernel. Many cereal grains contain B‐glucan, but significant quantities are found in barley (2%–20%) and oats (3%–8%), which is thought to provide the health benefits associated with both grains (Maheshwari, Sowrirajan, & Joseph, 2017).

Barley has been successfully incorporated into foods providing functional and nutritional benefits. Bacchetti et al. (2015) saw significant reductions in total cholesterol and low‐density lipoprotein‐cholesterol (LDL‐cholesterol) following barley–vegetable soup supplementation for 2 weeks. Barley β‐glucan incorporated into ready‐to‐eat cereal and fruit juice was well tolerated and reduced LDL‐cholesterol by 9% when supplemented at 3 g/day and 15% at 5 g/day in a population of hypercholesterolemic men and women (Keenan et al., 2007). Greater antioxidant capacities and vitamin E content were found in pita bread made with whole‐grain barley flour or pearled barley flour compared to pita bread with bread flour (Do, Muhlhausler, Box, & Able, 2016). Most studies to date have evaluated the health benefits of whole grain and minimally processed barley. However, there are opportunities to develop functional foods with heart health benefits using readily available, high‐volume, and low‐cost malted barley from the beer industry known as Brewers’ Spent Grain (BSG).

Brewers’ Spent Grains are the residual malted grains that remain after the mashing phase of beer production following removal of the liquid wort. Spent grains are the most abundant by‐product of beer manufacturing, representing around 85% of the total by‐products created with an estimated annual global BSG output of 30 million tons (Ktenioudaki et al., 2015; Mussatto, Dragone, & Roberto, 2006). Currently, craft breweries in the United States utilize BSG for animal feed, compost, alternative energy sources, and microorganism cultivation; however, BSG that is not utilized is deposited in landfills leading to loss of natural resources and landfill and greenhouse gas emission accumulation (Kitryte, Saduikis, & Venskutonis, 2015). With the U.S. craft beer industry producing over 24 million barrels of beer in 2016, equivalent to $23.5 billion in annual sales, and an estimated production growth of 34.6 million barrels by 2019 (Watson & Herz, 2017), the ability to recover BSG as a found food ingredient is an opportunity to reduce waste generated by the brewing industry.

Brewers’ Spent Grain typically includes the grain husk, pericarp, and endosperm fragments, and its composition can vary depending on the cereal grain bill, malting and mashing conditions, and adjuncts used during manufacturing (Fărcaş et al., 2015). However, all BSG has nutritional value from high levels of protein and dietary fiber with considerable levels of lipids, minerals, and polyphenols found mainly within barley, the most commonly used brewing grain (Waters, Jacob, Titze, Arendt, & Zannini, 2012). Many studies have shown the approximate chemical composition of BSG‐containing total carbohydrates (42%–60% w/w), fiber (19%–41% w/w), protein (15%–24% w/w), lignin (8%–28% w/w), fat (10% w/w), and ash (5% w/w) (Aliyu & Bala, 2011; Del Rio, Prinsen, & Gutierrez, 2013; Mussatto et al., 2006; Robertson et al., 2010; Santos, Jiménez, Bartolomé, Gómez‐Cordovés, & del Nozal, 2003). Additionally, BSG has a low glycemic score due to the low carbohydrate content from sugars being extracted during mashing for beer production.

The nutrition profile of BSG has led to its successful incorporation into different foodstuff for the development of new functional foods with specific health benefits. BSG flour has replaced 5%–30% of the total flour in many baked goods while maintaining consumer acceptance (McCarthy et al., 2013). Spent grains have been successfully incorporated into ready‐to‐eat baked and extruded snacks (Ainsworth, Ibanoglu, Plunkett, Ibanoglu, & Stojceska, 2007; Ktenioudaki et al., 2013; Stojceska, Ainsworth, Plunkett, & Ibanoglu, 2008), traditional breads and sourdough bread (Steinmacher, Nonna, Gasparetto, Anibal, & Grossmann, 2012; Waters et al., 2012), and breadsticks (Reis & Abu‐Ghannam, 2014) while significantly increasing in nutritional value via dietary fiber, protein, and mineral content in a dose‐depending fashion. The addition of 15% BSG into breadsticks more than doubled the content of dietary fiber (Ktenioudaki, Chaurin, Reis, & Gallagher, 2012). The addition of BSG can alter the odor, color, texture, and flavor profile of the product. Therefore, sensory testing is necessary to validate consumer acceptance of newly developed food containing BSG. Sensory‐test results indicated BSG at 10% in finished foods were highly acceptable (Ktenioudaki et al., 2013). Using this agro‐industrial by‐product as an ingredient in finished foods is appealing because it provides an opportunity to reduce waste created by the brewing industry while improving the nutritional content of food formulations.

To develop BSG‐containing foods while maintaining consumer acceptability, we need a better understanding of the factors that influence dietary behavior related to whole grain and BSG food intake. A consumers’ decision to purchase functional foods can be influenced by many factors, including familiarity with the product ingredients, brand loyalty, price, taste, and potential health benefits (Sook Chung et al., 2011). It has been shown that personal attitude can influence food choices and may predict willingness to consume a healthier diet (Tuuri, Cater, Craft, Bailey, & Miketinas, 2016).

Previous research has shown many barriers related to whole grain intake among adults including inability to identify whole grains, lack of knowledge in health benefits, negative sensory perception, or experiences such as taste and color, higher price, preparation and cooking time, and lack of availability (Kamar, Evans, & Hugh‐Jones, 2016; Kuznesof et al., 2012). Only 7.7% of U.S. adults consume the recommended 3–5 servings/day of whole grains (McGill, Fulgoni, & Devareddy, 2015; Reicks, Jonnalagadda, Albertson, & Joshi, 2014) with college‐aged adults (mean age = 20.5 years) reporting even lower intakes with an average of 0.58–0.68 servings/day (Burgess‐Champoux, Larson, Neumark‐Sztainer, Hannan, & Story, 2009). Particularly, college students fail to meet whole grain recommendations by consuming only 12% of the recommended minimum amount of three 1 oz. servings (Arts, English, Greene, & Lofgren, 2016; Ha & Caine‐Bish, 2011). To our knowledge, there are no studies that explore intake barriers that would inhibit willingness to consume BSG among college students.

Focus groups are viewed as, “flexible and cost‐effective method[s] for exploring attitudes, experiences, and responses” (Sofaer, 2002, p. 330) where human panelists are used as the instrument for data collection. The purpose of this exploratory study was to determine whole grain consumption patterns, perceptions of whole grains versus refined grains, interests, or expectations associated to BSG foods and concerns with consuming BSG foods among college students using focus group discussions. Additionally, these focus groups were used to gather input for product development stages of BSG foods that will serve as test foods for future diet interventions.

## 2 METHODS

### 2.1 Focus group discussions

The focus group is a form of qualitative research data collection used in early stages of product development to discuss and identify consumers’ needs, which is critical in creating foods that deliver preferable and unique benefits to consumers. The purpose of focus groups is to collect data in a social context where participants are allowed to examine their responses within the viewpoint of others. Using a grounded theory approach, this study utilized focus groups to identify the most important drivers of consumer choices, preferences, and expectations related to whole grain and Brewers’ Spent Grain food products, product purchasing decisions, and desired product attributes to guide BSG product development. Typically, a group of 8–10 participants are led in an open‐ended discussion by the moderator to gain access to consumer behaviors and perceptions in market research or sensory analysis, attitudes toward new food products or to define consumers’ preferences concerning product quality (Barlagne, Cornet, Jean‐Marc, Jean‐Louis, & Ozier‐Lafontaine, 2016).

### 2.2 Subjects

Participants were recruited through an open call email sent to all Texas Woman's University (Denton, Texas) undergraduate and graduate students in September 2016. The email informed potential participants of the study's purpose, focus group session dates, eligibility requirements, and incentives for participation. A 20‐dollar Target gift card and free food were incentives for participating in one of the focus group discussions. Participant eligibility included male and female undergraduate and graduate students aged 18–45 years able to consume gluten‐containing foods on a regular basis with no known food allergies or intolerances. Exclusion criteria included individuals with a food allergy or intolerance and women who were pregnant or lactating. Approval of the study was obtained from Texas Woman's University Institutional Review Board, Denton, TX before the study commenced. All participants were provided informed consent prior to participation, and signed consent was required for study participation. Participants were informed that all study results would be collected anonymously and the results would be provided upon request following completion of the study.

### 2.3 Procedure

All focus groups were held in a conference room on the university campus and consisted of a 60‐min session that was audio recorded and field notes taken. Upon entering the room, all participants were welcomed by the moderator and asked to fill out a Willingness to Eat Whole Grains questionnaire (Table 1) adapted from Tuuri et al. (2016) and a demographics questionnaire that included gender, age range, college major, and college classification questions. Participants were also asked if they consume grain‐based foods, review the nutrition facts panel, consider fiber content when purchasing foods, and rank the importance of food attributes.

**Table 1:** Willingness to eat whole grains questionnaire

Please completely fill in the appropriate circle with your response.					
How willing are you to eat the following foods?					
	Never eaten	Always eaten	Sometimes eaten	Sometimes willing	Always willing
Whole wheat bread	○	○	○	○	○
Whole grain granola bar	○	○	○	○	○
Whole wheat pasta	○	○	○	○	○
Whole grain tortilla	○	○	○	○	○
Whole grain pizza crust	○	○	○	○	○
Whole wheat bagel	○	○	○	○	○
Whole wheat muffin	○	○	○	○	○

*Note*

Each focus group participant filled out a Willingness to Consume Whole Grains questionnaire to determine which whole grain foods they would be willing to eat. Adapted from “Exploratory and confirmatory factory analysis of the willing to eat whole grains questionnaire: a measure of young adults’ attitudes toward consuming whole grain foods,” by G. Tuuri, M. Cater, B. Craft, A. Bailey, and D. Miketinas, 2016, *Appetite,* 105, p. 466.

All focus group participants were provided with nonmilled BSG, milled BSG flour, and snacks (pumpkin bread, peanut butter cookies, and granola bars) containing BSG. The nonmilled and milled BSG was provided for participants to visually inspect, and the snacks were provided for consumption; however, the participants were not informed the snacks contained BSG. Each group was led by a moderator while a co‐moderator assisted in the discussion by taking notes and collecting questionnaires. The moderator provided a brief introduction of the focus group topic and explained how the results would be used for future product development guideance.

Basic guidelines and rules were provided followed by obtaining signed consent from all participants. The moderator clarified there were no right or wrong answers and all participants were to respect differing points of view to ensure equal participation within the focus groups.

**Figure 1:**
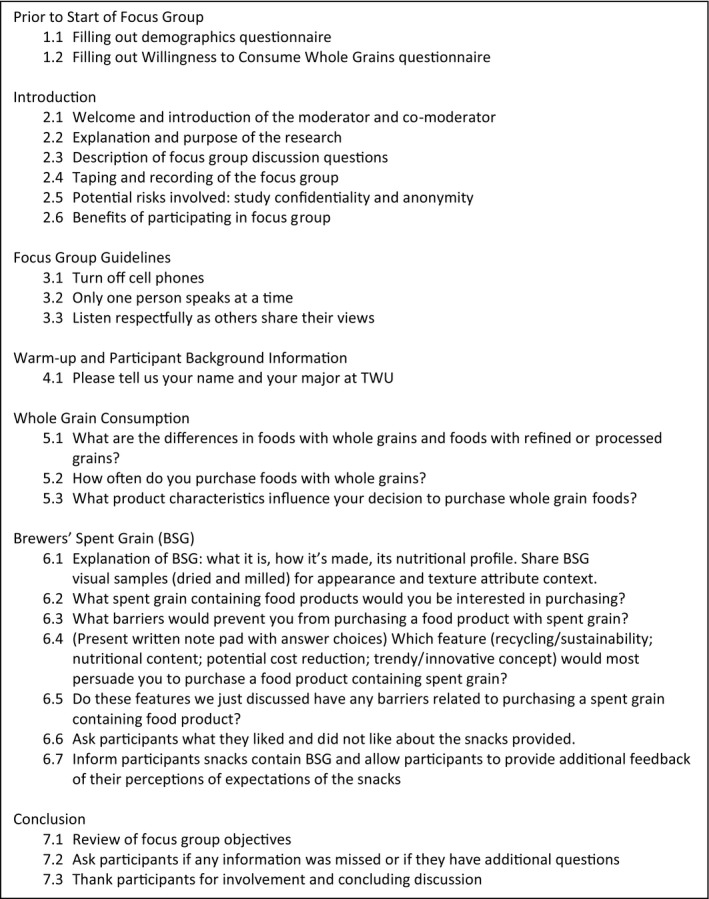
Semi‐structure interview guide used by the moderator to guide focus group discussions. Focus group discussions included seven open‐ended questions and one multiple‐choice question

Focus group discussion was guided using a semi‐structured interview guide (Krueger & Casey, 2015) with a series of seven open‐ended questions and one multiple‐choice question (Figure 1). The semi‐structure interview allowed participants the flexibility to discuss their thoughts and for the primary researcher to probe for clarification. The discussion focused on whole grain consumption, perceptions of nutritional value differences in whole grains and refined grains, and interest or barriers related to BSG purchasing and consumption. The focus group ended when the primary researcher deemed that all questions were thoroughly exhausted and participants had no remaining thoughts or input.

### 2.4 Data preparation and analysis

Each focus group audio recording was transcribed verbatim and analyzed using constant comparative analysis. Transcripts were organized into double‐spaced paragraph format with line numbers provided in the left margin to allow for quick location of quotes. Two researchers read each transcript a minimum of three times to get a general understanding of the entire dialog. Any overlapping and repetitive statements unrelated to the topic of interest were removed from the analysis.

Once all transcripts were read in the entirety, both researchers open coded each transcript to separate participant statements into “meaning units,” which can be one word or an entire paragraph, having one self‐contained idea or concept (Giorgi, 1985). Statements considered relevant to the research aims were treated as having equal significance. Meaning units were clustered into similar categories or themes that emerged using constant comparative analysis. To maintain rigor of the study design, both researchers met to debrief and discuss all meaning units and categories that emerged during data analysis. All themes were defined and described based on the experience being invested by all participants instead of individual experiences (Table 2).

**Table 2:** Focus group themes and respective definitions

Themes	Definitions
Concept of Health	Healthy is better for you because of the decreased number of ingredients resulting in the increase in quality. The negative association of healthy food tasting bad or lacking in flavor.
Sensory	Appearance/aroma/flavor/texture/noise influence preference for whole grains. Texture and taste considered most important attributes.
Experience with Brewers’ Spent Grain	Panelist's reactions to snacks provided after being informed Brewers’ Spent Grains were in foods. “Tastes like something my mom would make.” “I would totally buy this… if you made something like this.”
Consumer Education	What are Brewers’ Spent Grains? “I think having educational knowledge can push me into buying that product.” What needs to be done to increase familiarity of this product?
Marketing	Explains “why people buy what they buy.”
Cost	Healthy food costs more. College students are on a limited budget, which is the determining factor with whole grains purchased.
Whole Grain Purchasing Habits	Products purchased and the occurrence they were purchased in (daily, weekly, monthly, sporadic)
Whole Grain Product Experience	Critical in participant's continued purchase. Whole grains associated with feeling fuller.
Environment	Family/culture/life experiences influence our food choices.
Barriers of Brewers’ Spent Grain	Due to lack of consumer education.
Pop Culture Science	Language used by participants to describe foods when they weren't sure what the correct term was. Examples used by participants include: “organic,” “natural,” “foodie,” “gluten‐free,” “refiner,” and “vegetarian.”
Time	Amount of time participants give to shopping, preparation, and cooking of food.
Acculturation	Our taste palettes change or adapt over time.

*Note*

All thirteen themes and their respective definitions that emerged during focus group discussions in order of frequency.

## 3 RESULTS

### 3.1 Subject demographics

Six focus group discussions were conducted in fall 2016 and consisted of 34 women and three men with 29 of the participants in the 18–22 age group, five in the 23–30 age group, and three in the 31 and older age group. Focus groups were primarily comprised of female participants (92%) and with few male participants (8%). The participants were predominantly undergraduate students (89%) compared to graduate students (11%).

Frequency of each criterion was calculated from the Willingness to Eat Whole Grains questionnaire data and question 6.4 from the interview guide (Table 1). All questionnaire data were analyzed as frequency counts and presented as the percentage of participants who chose that criterion.

### 3.2 Willingness to eat whole grains

**Figure 2:**
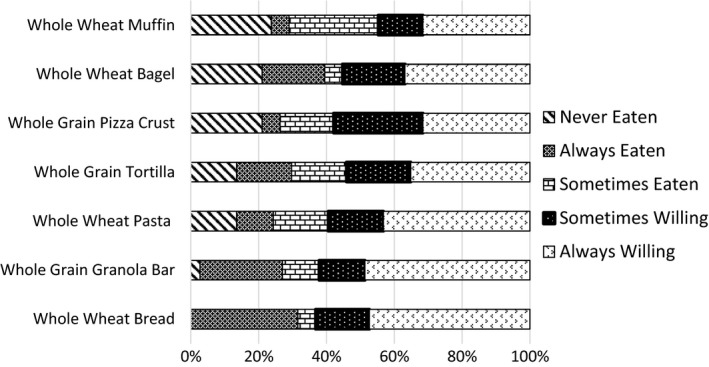
Results from Willingness to Eat Whole Grains questionnaire completed by all participants showing which whole grain products focus group participants are more willing to eat or already eat regularly. Results expressed as a percentage

Results from the Willingness to Eat Whole Grains questionnaire given to all focus group participants is shown in Figure 2. Whole wheat bread (31.58%) and whole grain granola bars (24.32%) were chosen as “always eaten” by participants more often than the other whole grain foods. Participants were also more willing to consume whole wheat bread and whole grain granola bars than the other whole grain foods polled. Participants had always eaten (31.58%) and were always willing (47.37%) to eat whole wheat bread, and participants had always eaten (24.32%) and were always willing (48.65%) to eat granola bars. Other products participants identified as being always eaten included whole grain tortillas (16.22%) and whole grain bagels (18.42%).

### 3.3 Focus group themes

Thirteen themes emerged from the coded transcripts. In order of most frequently discussed, these themes are as follows: Concept of Health, Sensory, Experience with Brewers’ Spent Grains, Consumer Education, Marketing, Cost, Whole Grain Purchasing Habits, Whole Grain Product Experience, Environment, Barriers of Brewers’ Spent Grain, Pop Culture Science, Time, and Acculturation. Each theme was defined to summarize the meaning units provided during focus group discussions (Table 2). Frequency counts and percentages are shown in Table 3 presenting the distribution of each theme from all focus group discussions. Concept of Health (26.36%), Sensory (21.77%), and Experience with Brewers’ Spent Grain (13.61%) represent nearly two‐thirds of the discussion's thematic content.

**Table 3:** Frequency counts and percentages of focus group themes

Theme	Frequency count	Frequency represented as percentage (%)
Concept of Health	155	26.36
Sensory	128	21.77
Experience with Brewers’ Spent Grain	80	13.61
Consumer Education	47	7.99
Marketing	44	7.48
Cost	34	5.78
Whole Grain Purchasing Habits	26	4.42
Whole Grain Product Experience	25	4.25
Environment	16	2.72
Barriers of Brewers’ Spent Grain	11	1.87
Pop Culture Science	9	1.53
Time	7	1.19
Acculturation	6	1.03

*Note*

The frequency count represents the number of times meaning units for each theme were discussed in all focus groups.

## 4 MAJOR THEMES

### 4.1 Concept of health

The most frequently discussed theme was Concept of Health, which described what the words “health” or “healthy” mean to participants. The words “health,” “healthy,” “nutrition,” and “nutritious” were commonly used when describing the differences in whole grain foods and foods containing refined grains. The definition of these words and the concept of health within focus groups were varied due to panelist subjectivity. Generally, participants believe healthier means the food is better for you and provides health benefits. The subjectivity of “health” is due to information presented to participants during life experiences and who or what they believed to be reputable sources of information such as family members, social media, food industry, doctors.

Participants all agreed that whole grains are healthier than refined grains but had mixed answers on why they believed this or could not provide an answer:

For as long as I can remember whole grains are better for you like when we were little we were taught the pyramid and grains were at the bottom, and we were supposed to eat so much of it. So I don't know why it's better, I'm not a nutrition major, but I'm sure whole grain is better for you.

Whole grains were thought to be less processed and more nutritious due to containing more nutrients such as fiber, protein, and vitamins along with reduced sodium and added sugars. Descriptive terms such as “organic,” “natural,” and “real” were used to convey a healthier status of whole grains compared to refined grains. One panelist felt whole grain products have more care or additional effort is used during development and manufacturing creating a healthier product:

There's like a little more care put into making it healthier.

It is possible this panelist associates health with products made locally or homemade from friends and family thus driving an emotionally perceived health benefit and higher quality to whole grain foods.

According to participants, refined grains are lacking in nutrients from components being stripped out during processing and have unhealthy ingredients added:

I watched something a short documentary where they strip the grain and then if it's whole wheat then it's not they didn't take all the good stuff off basically. That's my understanding, which is pretty limited, so I look for “whole” on the package.

There are negative connotations associated with refined grains because participants believe they are fake and contain unnecessary ingredients like added sugars, sodium, and artificial preservatives. Participants indicated they are looking for short, simple ingredient statements also known as “clean” ingredient statements without additives or chemicals. Several ingredients participants do not want on a healthy food ingredient statement are refined sugars, artificial sweeteners, and bleached flours. Long labels with ingredients that cannot be pronounced, or they are unfamiliar with are considered a “pharmacy label” on a “medicine bottle.”

### 4.2 Sensory

The second most discussed theme in the focus groups was Sensory (see Table 2), which focused on whole grain and refined grain product attributes. Appearance, aroma, flavor, texture, and noise are all attributes by which a food is perceived. Sensory plays an important role in consumer acceptance because positively and negatively perceived sensory aspects directly impact the overall liking of a food product. The predominant sensory attributes discussed by focus group participants were texture and taste followed by appearance. Participants received no prior sensory training and were considered untrained consumers.

Two groups of consumers emerged when discussing product attributes of whole grains and refined grains. Participants who regularly eat whole grains look for products darker in color with visible grains that are dense, so they can taste the grains and the crunch of the grains:

For me I like to look at the color. It's just the overall appearance of it makes me want to buy it. I feel like if it's darker for some reason I'll go it. The texture I don't mind if it's gritty or not. I like when the bread has the little nuts or grains in there.

Cause like bread there's lots of different whole grain bread. There's so many but I like my bread to have stuff like 7 grain, or I like to be able to see the grains and be able to see that and when I eat it I want to be able to eat and have my sandwich with the meat and be able to taste the crunch of the whatever's in there.

The whole grain consumer group believes refined grains, which they compare to Wonder^®^ bread, is too sweet and “looks unnatural” or “feels fake.” The refined grain consumer group had contradicting expectations for grain‐based products. This group indicated they are looking for products with a “soft and consistent” texture and appearance lacking seeds and nuts that has a sweeter taste:

Yeah like I'll buy wheat bread, and it will just sit there like the whole loaf and I'll never eat it cause I'm never in the mood and I'm always in the mood for white bread. I'm never in the mood for wheat bread cause there's like a big taste and texture difference. It just doesn't taste as good. I can't think why it doesn't taste as good. I think white bread is just softer and that's a big thing…

They believe whole grain products are too dense and have a drier mouthfeel than refined grains. Each of these groups explained if they observed a smooth and consistent appearance, they expected a smooth and consistent mouthfeel and would not purchase the product again if the appearance did not provide the same expected textural or mouthfeel experience and vice versa with products containing visible seeds and grains on the product's surface.

### 4.3 Experience with Brewers’ Spent Grain

Foods made with BSG were provided as snacks to participants during each focus group session; however, participants did not know BSG was used to prepare the foods. The snacks included cookies, sweet breakfast breads, and granola bars. Participants were not required to eat the snacks but were encouraged to taste and sample them. Two clear glass mason jars containing finely milled BSG and nonmilled BSG were also passed around during the focus group, so participants could see the grains’ appearance. Participants could open the jars and smell or touch the grain. The theme “Experience with Brewers’ Spent Grain” involved the participants’ reactions to seeing BSG in the mason jars and BSG‐containing foods they ate.

Participant's experiences with BSG provided during the focus groups elicited both positive and negative responses. When viewing and smelling BSG in the mason jars, participants felt the grains smelled “sweet,” “earthy,” “warm,” and “nutty.” Some participants felt the grains smelled “like home” related to the aroma of freshly baked homemade bread. Some participants associated the aroma and flavor with existing foods in the market like Grape‐Nuts^®^, Wheat Thins^®^, or graham crackers. The appearance to most participants was like whole grain flour, but to some it looked like sawdust or “hamster cage savings.” After foods containing BSG were eaten and participants were informed BSG was used in the recipes, they were encouraged to discuss their reactions to the snacks. Participants commented the foods were more “malleable” than expected, but there was a lingering fiber aftertaste. Sometimes when participants bit down, they could hear the bran crunch making the product texture feel “tough” at times. Participants liked that they could taste the grain:

You could smell the taste or something. Like I'm tasting it, and I'm like I taste the grain like I don't know how to explain it but I'm like oh this tastes healthy. It was definitely denser and I'm taking it off the roof of my mouth with my tongue. It's compact, but I liked it a lot. It was good. It was really moist.

### 4.4 Consumer education

The theme of “Consumer Education” focused on the importance of consumers being informed of the origin of BSG, that BSG is nonalcoholic, how it can be used in recipes and its nutritional content. Some participants felt the name “Brewers’ Spent Grain” is misleading because there is no beer or alcohol present, whereas others felt it is necessary to leave “brewers” in the name to explain its source. The unfamiliarity of BSG is a barrier that could prevent participants from purchasing foods containing the grain:

I think the barrier that I don't have any knowledge on it so for me it's like a foreign thing so I would be like I don't even know what that is so why would I buy it.

If I didn't know what spent grain was I would probably kinda be like meh looks good, maybe or maybe not, rather than I'm convinced. I have read about this product and know it's good and purchase it. I'm not saying I wouldn't buy it. I'm saying I think having educational knowledge can push me into buying that product.

Participants explained people needed to know more about the product, taste it, and see how it is used in recipes before they would make a purchase. Commercials, taste testing and sample recipes on product packaging were some of the methods suggested by participants to increase consumer awareness:

I think how to use it. Like if you buy the flour‐based product, how would you exactly use it except for baking. I think if you include recipes it would help. Probably adding recipes like on the back of certain products. That way with oatmeal like they give you the oatmeal raisin cookies recipe because personally if I saw it I wouldn't know what it is or what to use it for. I think knowledge of what you can make with it.

### 4.5 Marketing

Marketing in the food industry involves the promoting of foods that influences a person's decision to purchase or taste a food. Market research is used to explain why people buy what they buy. Common marketing concepts discussed by panelists included packaging design, front package labeling, the nutrition facts label, and the ingredient statement. Participants concluded BSG foods should have recyclable packaging and/or packaging made from recyclable materials. The package should be “natural looking” or have a “healthy look.” Health claims and marketing explaining the nutrition content is another important aspect to consider when designing the packaging:

The biggest thing for me is it needs to catch my eye if it has like stuff on the front letting me know it's high protein, high fiber. That's what's gonna get me to pick it up, turn it around and look at the nutritional content.

Additionally, participants suggested future BSG foods be sold in natural grocery store retailers like Whole Foods and Sprouts where health‐conscious consumers would be more likely to purchase it.

## 5 MINOR THEMES

The following themes combined represent approximately 23% of the focus group theme relative importance: Cost, Whole Grain Purchasing Habits, Whole Grain Product Experience, Environment, Barriers of Brewers’ Spent Grain, Pop Culture Science, Time, and Acculturation. Participants felt higher costs are associated with whole grain foods compared to refined grain foods, and price was a common deciding factor when choosing white bread over whole grain bread. Younger traditional college student participants were, “torn [by] wanting to eat healthy but not wanting to spend more money,” whereas older nontraditional college student participants with higher incomes were willing to pay more for higher quality and more nutritionally dense products. The theme Whole Grain Purchasing Habits explains what category and type of whole grain foods are purchased by the participants and the pattern in which the foods were purchased such as daily, weekly, monthly, or sporadically. Whole Grain Product Experience encompasses the reactions participants had after purchasing and eating whole grain foods. It was determined positive experiences will drive participants to subsequent purchases and negative experiences will prevent them from purchasing the product again. Experiences in life that have affected the participant's food choices or expectations explain the Environment theme. One participant explains, “I usually just go with what I know as far as what I've been brought up with and what I know like this is what we eat in my house…” The theme Barriers of Brewers’ Spent Grain is closely related to the theme Consumer Education because participants felt a lack of knowledge in BSG would prevent them from purchasing foods made with the grain.

Additional barriers include sensory aversions such as dark appearance, aroma, and textural differences from other grain‐based flours. Participants used buzz words when they were not aware of the scientifically accurate term or common terms used by the food industry. These participant terms were classified as Pop Culture Science. The theme of Time explained the amount of time participants were willing to give toward grocery shopping, food preparation, and cooking. Most participants purchased foods that were convenient, could be eaten on‐the‐go, and required little preparation. The last theme is Acculturation, which explains the acceptance of a food that is initially considered foreign and over time of continued exposure the taste palate will change or adapt and even develop an appreciation for the food. Participants explained acculturation of whole grain foods was necessary when switching from refined grains.

## 6 DISCUSSION

Brewers’ Spent Grain is the leftover malted barley grains from beer production, and while BSG compositions can vary, it always includes high levels of protein and dietary fiber with considerable levels of lipids, minerals, and antioxidants (Waters et al., 2012). The brewing industry creates significant amounts of BSG waste each year leading to landfill accumulation and generation of methane gas. Therefore, the opportunity to use BSG as an ingredient in food is of great interest. To do this, it is necessary to understand the drivers and barriers of consuming whole grain foods, and we can deduce these factors are similar to the ones influencing consumer BSG food choice and preference as BSG is derived from cereal whole grains. In this study, focus groups were used to explore knowledge, perceptions, and consumption of whole grains along with interests and barriers related to BSG foods to guide the development of new BSG food products.

Focus group participants recognized the term “whole grain” but many were unsure of the differences in the composition of whole grains compared to refined grains. Some participants did not recognize the term “refined grains,” and the term “processed grains” was used in its place for the remainder of the focus group discussion. Most perceived refined grains to have components removed making them less healthy compared to whole grains that contain all components of the original kernel. These findings support previous research by Magalis, Giovanni, and Silliman (2016) who found 75% of surveyed college students did not know the difference between a whole grain and refined grain. McMackin, Dean, Woodside, and McKinley (2012) also found many participants were uncertain about the differences between refined and whole grains eluding to the need for continued education on whole grains and the associated health benefits from regular consumption.

The Willingness to Eat Whole Grains survey conducted prior to focus group discussions indicated most participants were willing to consume various whole grain foods. Particularly, participants were more willing to eat foods such as bread and granola bars over other food items. This was also seen during focus group discussions as participants listed bread and granola bars more often as whole grain food they commonly purchased or consumed. Similarly, Ha and Caine‐Bish (2011) found granola bars and ready‐to‐eat cereals comprised 58% of total whole grain intake among college students. This study's findings may reflect the participant's familiarity with these products containing or being made with whole grains, while other whole grain products may not have been reported or were incorrectly reported such as with honey wheat breads that some participants believed contained 100% whole wheat flour. When developing new BSG baked goods, it is important to begin the development process creating products that are already recognized and well accepted by consumers such as breads, granola bars, and cereals.

The top three themes Concept of Health, Sensory, and Experience with BSG provided more than 60% of the focus group's discussion frequency. Concept of Health dealt with the healthiness perceptions of participants who believed whole grains are healthier or convey more health benefits than refined grains. Generally, participants did not know the difference in grain composition between whole grain and refined other than acknowledging components have been removed. It was understood that whole grains contain the whole intact grain, but it was not known why the nutritional composition of whole grain provides health benefits compared to refined grains such as bran and germ removal during milling. Additionally, most participants were unaware refined grains such as enriched flour is fortified to add back missing vitamins and minerals removed during processing. There are various beliefs focus group participants had that impacted their view of the nutritional value of a product including processing or refinement, ingredient statement length, and health claims such as organic, all natural, or non‐Genetically Modified Organisms (GMO). These findings are supported by previous research showing consumers considered natural functional foods as healthy compared to nonnatural functional foods (Teuber, Dolgopolova, & Nordström, 2016).

The theme of Sensory explains product attributes driving participant's acceptance or negative experience with a food product. The predominate attributes discussed by focus group participants were texture, taste, and appearance. The most significant barrier to whole grain intake is considered sensory properties of whole grains (Arvola et al., 2007; Dewettinck et al., 2008). Taste is considered one of the most important sensory attributes in functional foods because most consumers will not compromise on taste for health benefits (Teuber et al., 2016). It was found that hereditary consumers who are acculturated and grew up consuming whole grains prefer the darker color, visible grains, earthy taste, and fibrous texture of whole grain foods and are willing to pay more for them. Recently, Magalis et al. (2016) found white rice and pasta were liked significantly more than their whole grain counterparts, which were less familiar to the college student participants. Therefore, it is important to establish hereditary whole grain consumers as a market segment for new product development of whole grain foods and BSG foods.

Nonmilled BSG, milled BSG flour, and snacks containing BSG were provided to all participants and the discussion that followed generated the theme, Experience with BSG. Participants use similar terms to describe BSG samples and snacks including “sweet,” “warm,” “nutty,” and “baked bread.” These terms can be used to market the flavor profile of BSG foods. Some participants felt there was a lingering fiber aftertaste, which is likely from the bran and will need to be masked or diluted when developing new BSG foods. Texture and particle size of BSG is important because participants noted pieces were stuck in their teeth after eating BSG granola bars and some mentioned the peanut butter cookies had grainy pieces that were noticeable but not undesirable. Guo, Du, Zhang, Zhang, and Jin (2014) conducted sensory evaluations on BSG‐enriched biscuits and found that as the BSG particle size decreased, the sensory score became higher. Therefore, when developing new baked goods with BSG, it will be necessary to finely mill the flour to achieve smaller and more malleable particles.

The theme of Consumer Education explained that focus group participants consider unfamiliarity with BSG a barrier. Marketing BSG to educate potential consumers on the origin of BSG and its attributes: nonalcoholic, high protein, high fiber, lower‐glycemic index value, and environmentally friendly nature of BSG. Participants discussed sampling BSG is necessary to have an experience and establish beliefs and attitudes toward BSG. Dewettinck et al. (2008) explain food choice depends on a consumer's beliefs and attitudes toward a food with beliefs being external cognitive knowledge linking a product's attributes, benefits, and objectives, whereas attitudes are the internal feelings to a product's attributes. Determinants of perceived food quality are socio‐demographic factors (age, gender, income), food properties (sensory, health, safety), and environmental factors (price, marketing, labeling). For consumers to develop perception of food quality, they must be willing to try the food regardless of preconceived beliefs. Studies have found various nutrition educational interventions can increase consumer knowledge and influence their willingness to eat healthier food choices thus improving their overall dietary‐intake (Shahril, Wan Dali, & Lin Lua, 2013) or increase their whole grain intake (Ellis et al., 2005). Arts et al. (2016) found whole grain consumption was increased nearly 40% in college students following a benefit‐based text messaging intervention. Therefore, nontraditional electronic marketing and social media campaigns can provide BSG education to many consumer groups and specifically younger generations.

Minor themes worth noting involve purchasing habits and time allowed for shopping, preparation, and cooking. A common trend of convenient, on‐the‐go food requiring little preparation was discussed throughout all focus groups. These findings may reflect the younger age range of participants and their fast‐paced lifestyles. Many participants agreed healthy and filling BSG snack foods that could be eaten between classes would most influence their decision to sample and purchase BSG.

There are several study limitations due to the use of a convenience sample. The majority of participants were between 18 and 22 years of age, and younger consumers are typically less interested in foods with health benefits because they have not experienced any significant health problems (Bruschi, Teuber, & Dolgopolova, 2015). The majority of the focus group participants were female; therefore, results may represent the viewpoints of a female consumer better than a male consumer. In addition, college students are not considered the ideal whole grain focus group population because research indicates they consume less than one serving of whole grains per day (Rose, Hosig, Davy, Serrano, & David, 2007). Finally, participants may have reported products they believed to be whole grain but were not or underreported whole grain products they were unaware of.

## 7 CONCLUSIONS

To our knowledge, there are no studies that have explored the perceptions and barriers of BSG consumption. Therefore, we conducted a focus group study to determine consumer perceptions of whole grains compared to processed grains, concerns with consuming BSG foods, and foods consumers would want to eat that contain BSG. Thirteen themes emerged from focus group discussions with Concept of Health, Sensory, and Experience with BSG being the top three most discussed themes representing nearly two‐thirds of the discussion's thematic content. Study findings show focus group participants believe whole grains are more healthful compared to refined grains due to a lack of nutrients. The two consumer groups, hereditary whole grain consumers and hereditary refined grain consumers, emerged from the Sensory theme. Hereditary whole grain consumers should be used as future BSG test subjects because they are familiar with and acculturated to whole grain product attributes, which will help reduce sensory barriers associated with BSG. Educating consumers on the sensory attributes, potential health benefits, and environmental benefits of BSG will be necessary to help overcome the unfamiliarity barrier participants discussed. Overall, most participants like the BSG foods provided during focus groups. However, a few noted darker appearances and lingering fiber aftertaste along with particulates from the BSG bran. Masking the bran flavor and texture with additional textures from seeds and nuts or dried fruit may help improve the overall product texture and reduce the aftertaste of fiber in future product development stages. Future studies will involve the development of baked goods such as breads, granola bars, or cereal as these are well‐recognized and commonly consumed products containing whole grains. Sensory testing will follow product development to evaluate consumer acceptance of the baked goods containing varying percentages of BSG to determine the maximum allowable concentration acceptable to consumers.

## CONFLICT OF INTEREST

None declared.

## ETHICAL REVIEW

This study was approved by the Institutional Review Board of Texas Woman's University.

## INFORMED CONSENT

Written informed consent was obtained from all focus group study participants.
